# Lipopolysaccharide Challenge Reveals Hypothalamic-Pituitary-Adrenal Axis Dysfunction in Murine Systemic Lupus Erythematosus

**DOI:** 10.3390/brainsci8100184

**Published:** 2018-10-04

**Authors:** Grace S. Pham, Keisa W. Mathis

**Affiliations:** Department of Physiology and Anatomy, University of North Texas Health Science Center, Fort Worth, TX 76107, USA; grace.pham@live.unthsc.edu

**Keywords:** neuroimmune, vagus nerve, autoimmune disease, inflammation, corticosterone, cytokines

## Abstract

Crosstalk between the brain and innate immune system may be dysregulated in systemic lupus erythematosus (SLE), a chronic autoimmune disease that presents with dysautonomia and aberrant inflammation. The hypothalamic-pituitary-adrenal (HPA) axis is an endogenous neuro-endocrine-immune pathway that can regulate inflammation following activation of vagal afferents. We hypothesized that chronic inflammatory processes in SLE are in part due to HPA axis dysfunction, at the level of either the afferent vagal-paraventricular nuclei (PVN) interface, the anterior pituitary, and/or at the adrenal glands. To study this, we challenged female control and SLE mice with lipopolysaccharide (LPS) and measured c-Fos expression as an index of neuronal activation, plasma adrenocorticotrophic hormone (ACTH) as an index of anterior pituitary function, and plasma corticosterone as an index of adrenal function. We found that c-Fos expression in the PVN, and plasma ACTH and corticosterone were comparable between unchallenged SLE and control mice. PVN c-Fos was increased similarly in control and SLE mice three hours after LPS challenge; however, there were no changes in plasma ACTH amongst any experimental groups post inflammatory challenge. Plasma corticosterone was markedly increased in LPS-challenged SLE mice compared to their vehicle-treated counterparts, but not in controls. Paradoxically, following LPS challenge, brain and spleen TNF-α were elevated in LPS-challenged SLE mice despite heightened plasma corticosterone. This suggests that, despite normal c-Fos expression in the PVN and activation of the HPA axis following LPS challenge, this cumulative response may not adequately defend SLE mice against inflammatory stimuli, leading to abnormally heightened innate immune responses and peripheral inflammation.

## 1. Introduction

**Figure 1:**
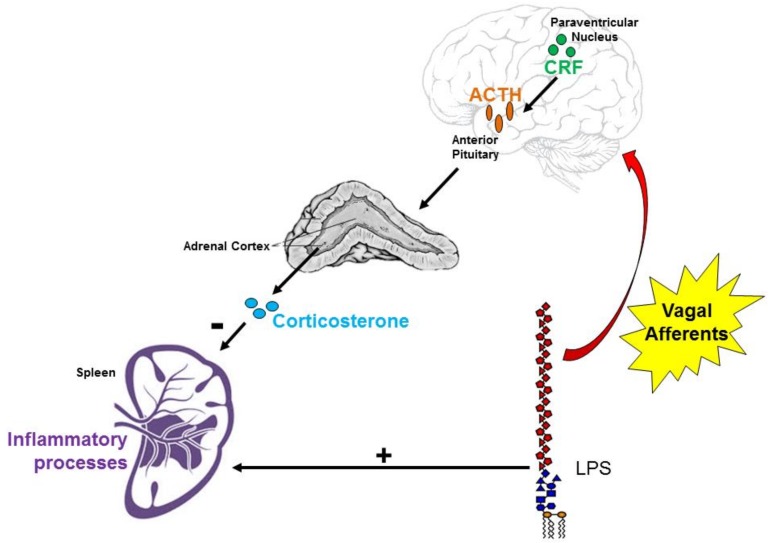
The vagus nerve is a conduit between the brain and immune system. Vagal afferents transmit information regarding peripheral inflammatory processes (such as lipopolysaccharide (LPS) challenge, which also induces the release of pro-inflammatory cytokines by splenic macrophages and other innate immune cells) to the nucleus tractus solitarius. This causes corticotropin releasing factor (CRF)-releasing parvocellular neurons in the paraventricular nucleus (PVN) of the hypothalamus to prompt adrenocorticotrophic hormone (ACTH) release from the anterior pituitary. ACTH from the bloodstream then binds to receptors in the zona fasciculata of the adrenal cortex to induce corticosterone release. Plasma corticosterone inhibits initiation of the inflammatory cascade.

Systemic lupus erythematosus (SLE) is a chronic autoimmune disease characterized by dysautonomia in the form of decreased vagal tone and aberrant chronic inflammation [1]. Due to this combination of features, the ability of the brain to regulate immune responses may be impaired in SLE. While the adaptive immune system is more commonly associated with SLE, due to the production of pathogenic autoantibodies, the innate immune system may also contribute to the pathogenesis of SLE. For instance, endogenous nucleic acids may activate toll-like receptors [2], and antigen presentation of self-DNA complexes by neutrophils to dendritic cells may exacerbate the disease course [3]. Neuroimmune interactions regulate the activity of both the innate and adaptive immune systems and their dysfunction may exacerbate autoimmune disease processes. The hypothalamic-pituitary-adrenal (HPA) axis is an endogenous neuro-endocrine-immune pathway that is commonly associated with psychological stress, but is also activated by vagus nerve afferents that are, in turn, activated by pro-inflammatory cytokines and bacterial products, such as lipopolysaccharide (LPS) (Figure 1) [4]. LPS directly stimulates the innate system via toll-like receptor 4 and promotes the transcription of IL-1 and TNF-α by immune cells [5]. LPS [6] and IL-1 [7,8] have been shown to induce afferent vagus nerve firing that then initiates HPA axis activation, beginning with parvocellular neuronal activation in the paraventricular nuclei of the hypothalamus, which leads to corticotropin releasing factor (CRF) release. CRF induces neurons in the anterior pituitary to release adrenocorticotrophic hormone (ACTH) into the bloodstream. ACTH binds to its receptors in the zona fasciculata of the adrenal cortex to prompt cortisol release (corticosterone in rodents). Cortisol inhibits both innate [9] and adaptive [10] immune processes via attenuation of the inflammatory cascade [9] and by shifting the inflammatory milieu from a pro-inflammatory Th1 to an anti-inflammatory Th2 profile [10], respectively. Cortisol and glucocorticoids may favor this shift to type 2-mediated anti-inflammatory responses by down-regulating pro-inflammatory Th17 cells [11]. Glucocorticoids also specifically down-regulate IL-1-mediated inflammatory events by upregulating naturally-occurring IL-1 inhibitors, such as IL-1 receptor antagonist and IL-1 receptor II [12]. Unlike other autoimmune diseases that are characterized by prevalent upregulations of Th1 and Th17 cells, such as multiple sclerosis and rheumatoid arthritis [13], Th1-, Th2-, and Th17-mediated events seem to cooperate in the development of lupus [14,15]. Taken together and on the basis of prevalent dysautonomia and chronic peripheral inflammation, we hypothesize that HPA axis dysfunction in SLE may lead to aberrant innate immune system activity.

HPA axis responses in SLE patients and mouse models are indeed dysregulated compared to those of their healthy counterparts [16,17,18]. Intuitively, this makes sense because SLE is an autoimmune disease with heightened peripheral inflammation, and deficits within the effector system that releases cortisol (or corticosterone in rodents), an anti-inflammatory steroid hormone, may contribute to chronic inflammation in SLE. Clinical studies have yielded mixed results when it comes to baseline cortisol, with some reporting decreased baseline cortisol [19] and others reporting comparable serum cortisol compared to healthy subjects [16,20]. In response to insulin-induced hypoglycemia, which is expected to activate the HPA axis due to the brain sensing decreased plasma glucose, there is an attenuated plasma cortisol or corticosterone response in SLE [16,17]. Others have shown that direct electrical stimulation of the vagal afferents, which is known to activate the HPA axis [21], leads to interleukin (IL)-1β mRNA expression in the brain [21] and increased plasma corticosterone [22], supporting this connection. On the basis of this relationship, one might expect dysfunction of the vagal afferent-HPA axis interface in SLE. 

While HPA axis dysfunction is present in SLE, it is unclear whether this is due to decreased afferent vagus nerve sensitivity to inflammatory molecules (such as LPS), or whether dysfunction is present further downstream in the HPA axis, such as at the adrenal gland. We, therefore, hypothesized that female SLE mice would exhibit decreased c-Fos expression, an index of neuronal activation, in the paraventricular nucleus of the hypothalamus, as well as decreased ACTH and corticosterone responses, following LPS challenge. We additionally anticipated that basal plasma ACTH and corticosterone would be attenuated in unchallenged SLE mice compared to control strains, indicating a hypoactive HPA axis in SLE. Lastly, we expected that brain and spleen inflammation would be higher in SLE mice compared to controls, due to inadequate HPA axis activity, so, to investigate this outcome, we measured brain and spleen pro-inflammatory cytokine expression following an LPS challenge.

## 2. Materials and Methods

### 2.1. Animals

Female *NZBWF1*, *NZW/LacJ*, and *C57/Bl6J* mice were obtained from Jackson Laboratories. *NZBWF1* mice, a well-established murine model of SLE [23,24,25,26,27,28], that had an albuminuria of ≥300 mg/dL for two consecutive weeks, were used in this study under the rationale that these mice with aggravated disease course would display the greatest amount of HPA axis dysfunction, and yield larger differences compared to the *NZW* and *C57/BL6J* control strains. *NZW* mice are one of the parental strains of *NZWBF1* mice and exhibit mild autoimmunity, and were used as controls due to their similar lineage, yet lack of explicit SLE-like symptoms. *C57* mice were added as an alternate control strain as they do not share lineage with *NZBWF1* mice. All animal studies were approved by the University of North Texas Health Science Center’s Institutional Animal Care and Use Committee (IACUC) and were in accordance with National Institutes of Health (NIH) Guide for the Care and Use of Laboratory Animals.

### 2.2. Acute LPS Challenge

Animals were brought up to the laboratory space at 9:30 and allowed to habituate for thirty minutes. At 10:00, LPS (1 mg/kg dissolved in normal saline as vehicle [29]), or vehicle, was injected intraperitoneally at a total volume of 0.1 mL. Mice remained in the laboratory space until euthanasia at 13:00. Three hours following the induction of acute inflammatory stress, anesthetized mice were transcardially perfused with 10 mL of 2% heparinized saline, then 10 mL of 4% paraformaldehyde in PBS. Brains were harvested and remained in 4% paraformaldehyde overnight.

### 2.3. c-Fos and CRF Immunohistochemistry

After paraformaldehyde fixation, brains were kept in 30% sucrose dissolved in PBS for at least three days, until fully dehydrated. Brains were sectioned on a cryostat at 30 μm sections and kept in cryoprotectant until staining for immunohistochemistry as previously described [30,31]. Brain sections were washed with PBS, then incubated with rabbit anti-c-Fos primary antibody (SySy, Göttingen, Germany, 1:2000) dissolved in PBS diluent. A donkey anti-rabbit biotinylated secondary antibody (Vector Labs, Burlingame, CA, USA; 1:10,000) was used along with A + B Vector staining and DAB to visualize c-Fos expression. A guinea pig anti-CRF primary antibody (Peninsula, San Carlos, CA, USA; 1:1000) was then used along with a goat anti-guinea pig AlexaFluor 488 secondary antibody (Fisher, Hampton, NH, USA; 1:10,000) to confirm the presence and LPS-mediated activation of CRF-secreting parvocellular neurons within the paraventricular nuclei. Cells were imaged on an Olympus fluorescence microscope and counted manually on NIH ImageJ software [30].

### 2.4. Plasma Corticosterone and Adrenocorticotrophic Hormone (ACTH)

A separate subset of LPS-challenged SLE and control mice were anesthetized, and a terminal blood sample was taken 3 h post, in which plasma corticosterone and ACTH were later measured via commercial ELISA kits (Enzo, Farmingdale, NY, USA, and Phoenix Pharmaceuticals, Burlingame, CA, USA, respectively).

### 2.5. Brain and Spleen Cytokines

Brains and spleens were harvested from the subset of mice that were not used for c-Fos and CRF immunohistochemistry, then flash-frozen in liquid nitrogen and stored at −80 °C. Tissues were weighed and homogenized with 8 times their weight of RIPA buffer plus protease inhibitors. Western blotting was performed with primary antibodies against tumor necrosis factor (TNF)-α and IL-1β (Santa Cruz, Dallas, TX, USA; 1:250), and secondary antibodies conjugated to HRP (Rockland, Limerick, PA, USA; 1:5000) as previously described [28]. TNF-α was quantified at both the 26 and 51 kDa bands to gauge relative amounts of the transmembrane and active trimeric isoforms [32], respectively. Blots were imaged on a ChemiDoc imager (BioRad, Hercules, CA, USA) and analyzed using ImageLab software (BioRad, Hercules, CA, USA), with the bands of interest normalized to total lane protein using the stain-free quantification method, as previously reported [28].

### 2.6. Statistical Analysis

All data are calculated as mean ± standard error of the mean (SEM) and statistical analysis were performed using SigmaPlot 11.0 (Systat, Richmond, CA, USA). Statistical differences (*p* < 0.05) between multiple groups were determined by two-way ANOVA followed by a Holm-Sidak post-hoc test, as specified in the accompanying figure legend. As *C57* mice were used as an alternate control strain for vehicle treatment, a *t*-test was used to compare unchallenged SLE and *C57* groups.

## 3. Results

### 3.1. SLE Mice had Elevated Anti-dsDNA Autoantibody Titer Compared to NZW and C57/Bl6 Mice

**Figure 2:**
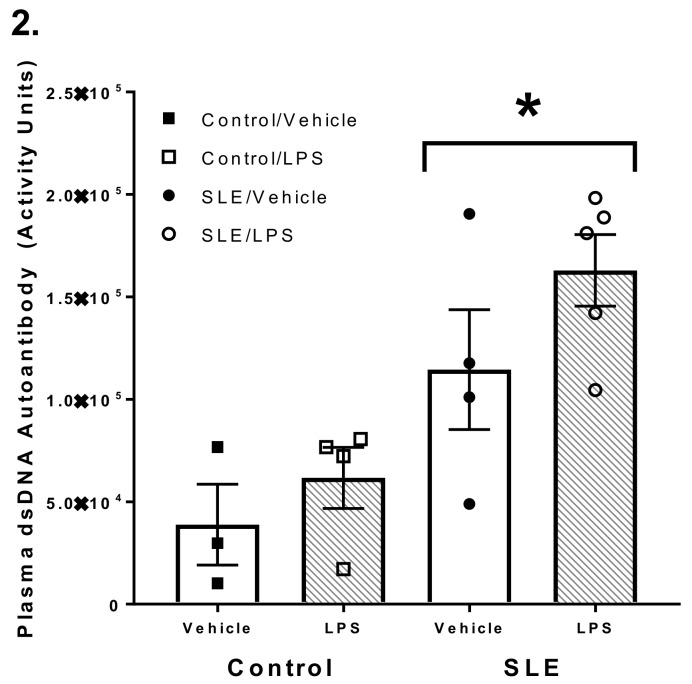
Systemic lupus erythematosus (SLE) mice have elevated plasma anti-dsDNA autoantibodies compared to *NZW* and *C57* control mice, regardless of treatment group. All values are reported as mean ± SEM, and the results of the two-way ANOVA indicated a significant difference between control and SLE mice. * *p* SLE/Vehicle and SLE/ lipopolysaccharide (LPS) vs. Control/Vehicle.

Plasma anti-dsDNA autoantibody titer is a commonly accepted index of disease severity in both clinical and experimental lupus. A *t*-test revealed there was a significant difference in plasma dsDNA autoantibody between SLE and *C57* female mice (1.1 × 10^5^ ± 2.9 × 10^4^ vs. 5.7 × 10^3^ ± 1.3 × 10^3^ activity units; data not shown; *n* = 3 mice/group; *p* = 0.036). SLE mice also had greater plasma activity of these autoantibodies compared to *NZW* mice (1.1 × 10^5^ ± 2.9 × 10^4^ vs. 3.9 × 10^4^ ± 2.0 × 10^4^, *p* < 0.001) (Figure 2). Plasma dsDNA autoantibody activity did not change 3 h post-LPS challenge in SLE or control mice.

### 3.2. LPS Challenge Elicits Similar Parvocellular Paraventricular Nuclei Activation between SLE and Control Mice

**Figure 3:**
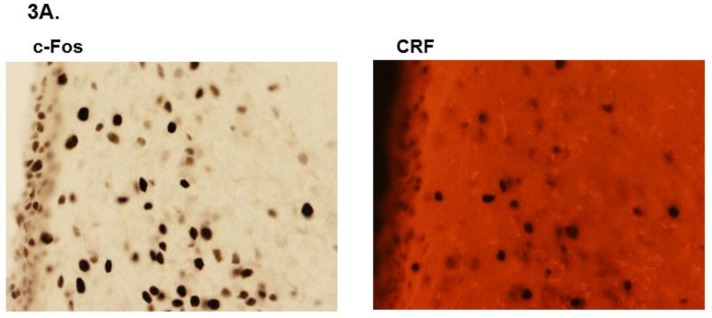

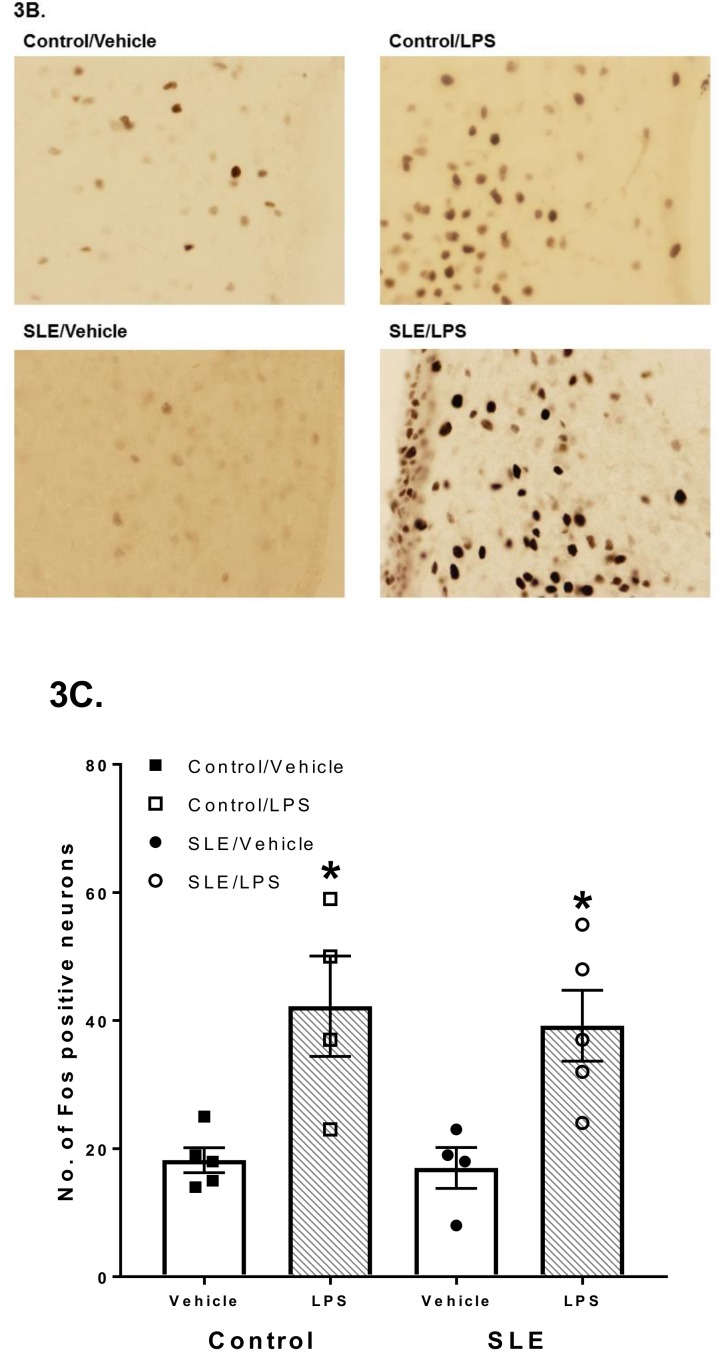
(**A**) Examples of c-Fos and corticotropin-releasing factor (CRF) staining in the paraventricular nucleus (PVN) parvocellularis. As vasopressin-secreting neurons also comprise the parvocellular region of the PVN, CRF staining was used to verify the presence and activation of CRF-secreting parvocellular neurons in the PVN. (**B**) Representative images of the paraventricular nucleus taken at 40× from systemic lupus erythematosus (SLE) and control mice. (**C**) Quantitative representation of positive cell counts between lipopolysaccharide (LPS)-treated SLE and control mice. All values are reported as mean ± SEM, and the results of the two-way ANOVA indicated significant differences between LPS-challenged SLE and control mice compared to their vehicle-treated counterparts. * *p* SLE/LPS vs. SLE/Vehicle and *p* Control/LPS vs. Control/Vehicle.

Following intraperitoneal injection of vehicle, paraventricular c-Fos expression did not differ between SLE and *C57* mice (17.0 ± 3.2 vs. 14.2 ± 2.9 cells/field; data not shown; *p* = 0.537). Similarly, there were no differences in c-Fos expression between SLE and control (*NZW*) mice (17.0 ± 3.2 vs. 18.2 ± 1.9 cells/field, *p* = 0.867). LPS increased c-Fos neuronal expression in both SLE and control mice compared their vehicle-treated counterparts (39.2 ± 5.5 vs. 17.0 ± 3.2 cells/field, *p* = 0.007 for SLE; 42.3 ± 7.8 vs. 18.2 ± 1.9 cells/field, *p* = 0.004 for controls) (Figure 3C), and by the same magnitude (39.2 ± 5.5 vs. 42.3 ± 7.8 cells/field, *p* = 0.672).

### 3.3. LPS has Differential Effects on HPA Axis Hormones in SLE Mice

**Figure 4:**
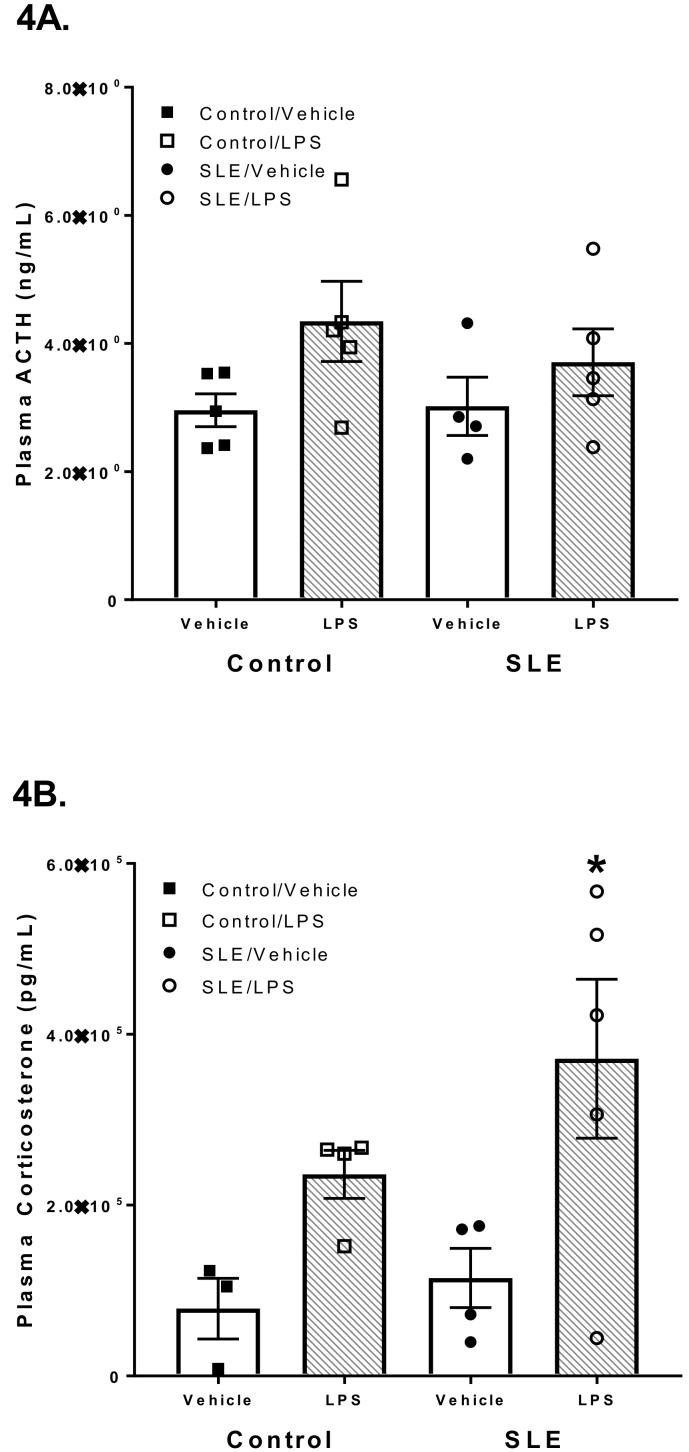
(**A**) Lipopolysaccharide (LPS) challenge does not affect plasma adrenocorticotrophic hormone (ACTH) concentration in systemic lupus erythematosus (SLE) and control mice. (**B**) LPS challenge provokes a significant increase in plasma corticosterone in SLE mice. All values are reported as mean ± SEM, and the results of the two-way ANOVA indicated a significant difference between LPS-challenged SLE mice and their n counterparts. * *p* SLE/LPS vs. SLE/Vehicle.

Following vehicle injection, plasma ACTH did not differ between SLE and *C57* mice (3.0 ± 0.5 vs. 3.7 ± 0.4 ng/mL; *p* = 0.378, data not shown), nor SLE and control (*NZW*) mice (3.0 ± 0.5 vs. 3.0 ± 0.3 ng/mL; *p* = 0.913; Figure 4A). Plasma ACTH also did not change following LPS challenge in both SLE and control mice (3.0 ± 0.5 vs. 3.7 ± 0.5 and 3.0 ± 0.3 vs. 4.3 ± 0.6 ng/mL, *p* = 0.393, respectively).

Plasma corticosterone was not different in SLE mice compared to *C57* mice (1.1 × 10^5^ ± 3.5 × 10^4^ vs. 4.7 × 10^4^ ± 2.0 × 10^4^; *p* = 0.348, data not shown). Similarly, plasma corticosterone was not different in SLE mice compared to *NZW* controls (1.1 × 10^5^ ± 3.5 × 10^4^ vs. 7.9 × 10^4^ ± 3.6 × 10^4^; *p* = 0.613; Figure 4B) LPS-challenged SLE mice had increased plasma corticosterone compared to vehicle-treated SLE mice (3.7 × 10^5^ ± 9.3 × 10^4^ vs. 1.1 × 10^5^ ± 3.5 × 10^4^; *p* = 0.009). By contrast, plasma corticosterone was not significantly altered in LPS-challenged control mice compared to their vehicle-treated counterparts (2.4 × 10^5^ ± 2.8 × 10^4^ vs. 7.9 × 10^4^ ± 3.6 × 10^4^; *p* = 0.152; Figure 4B).

### 3.4. LPS Alters Brain and Splenic Pro-Inflammatory Cytokine Expression

**Figure 5:**
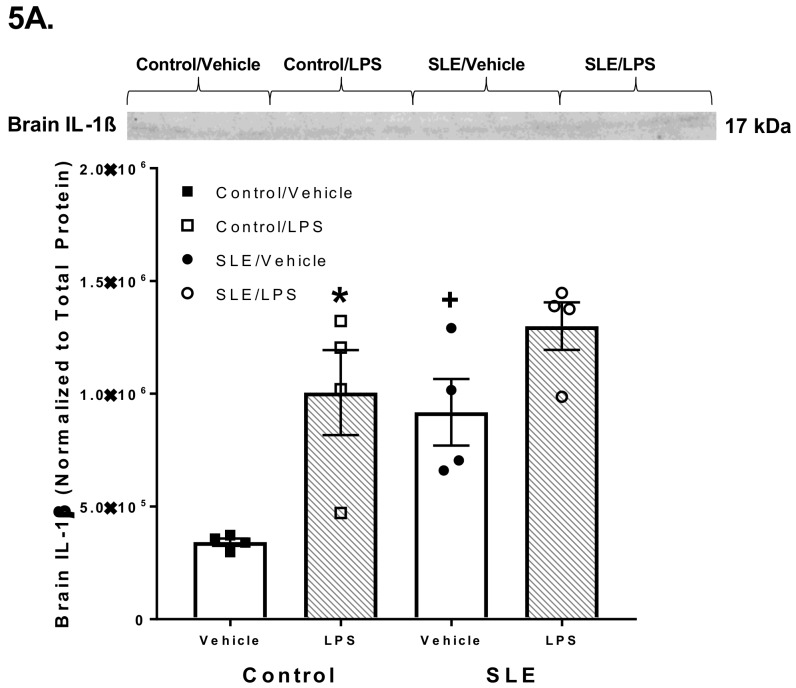

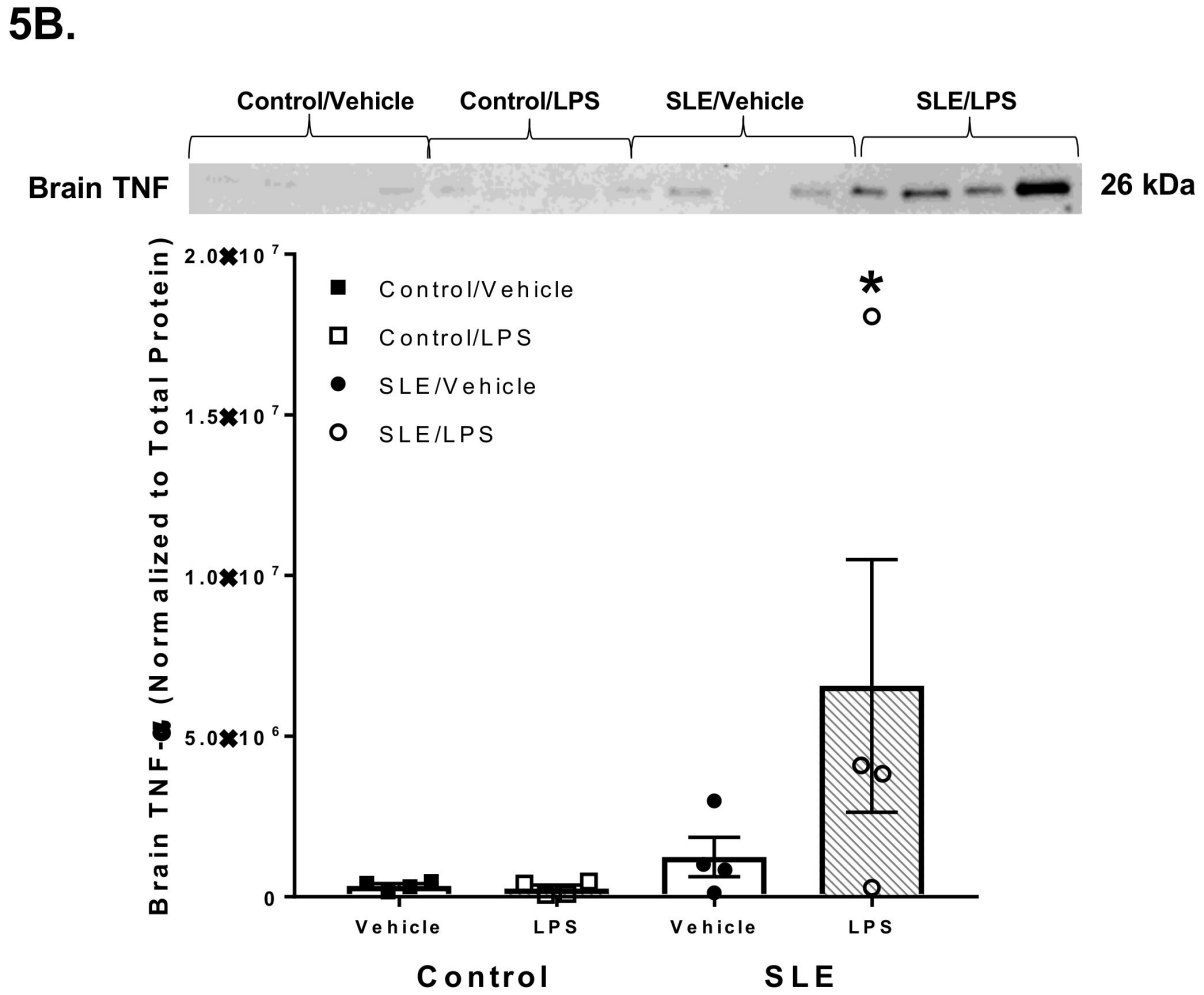

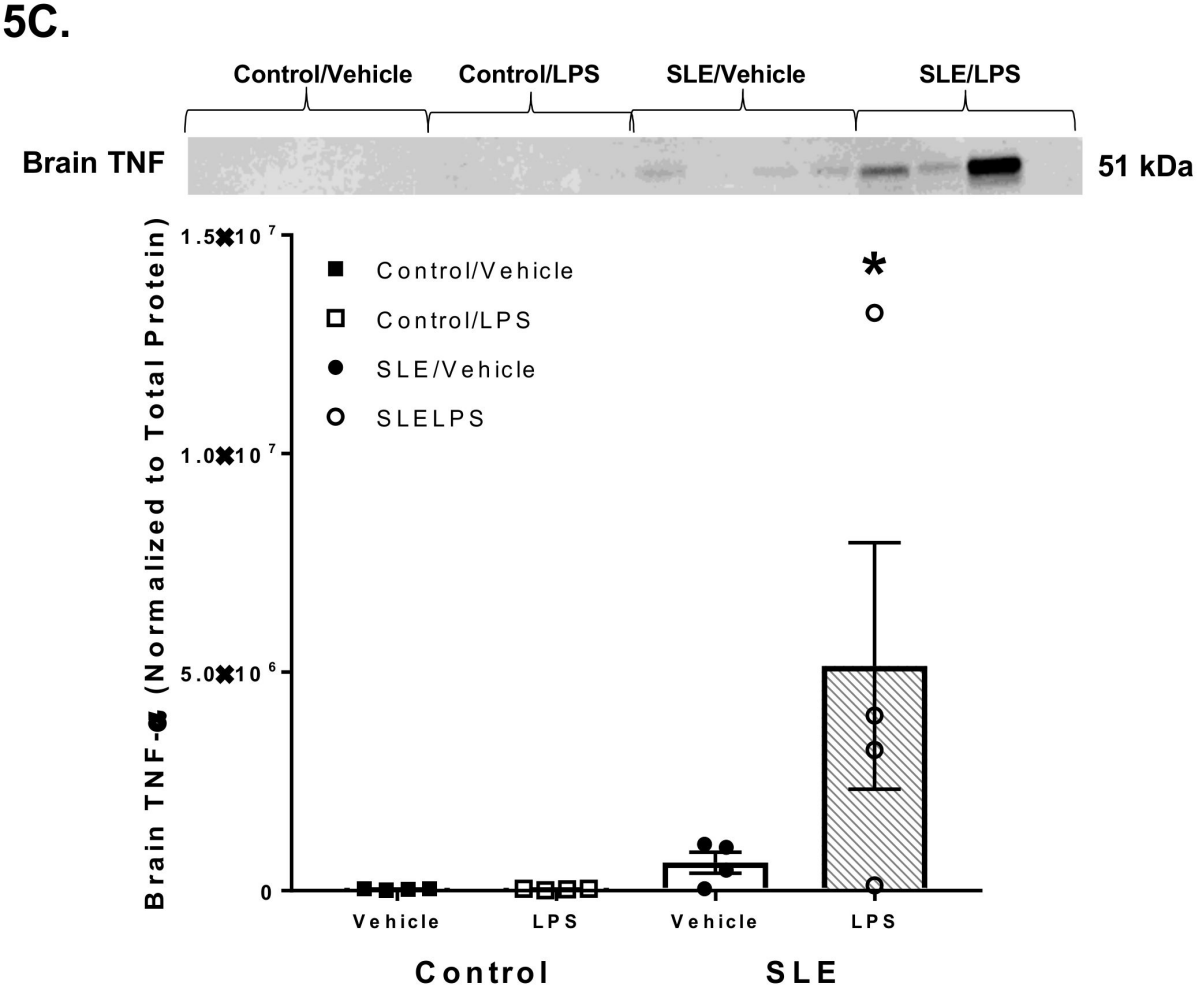

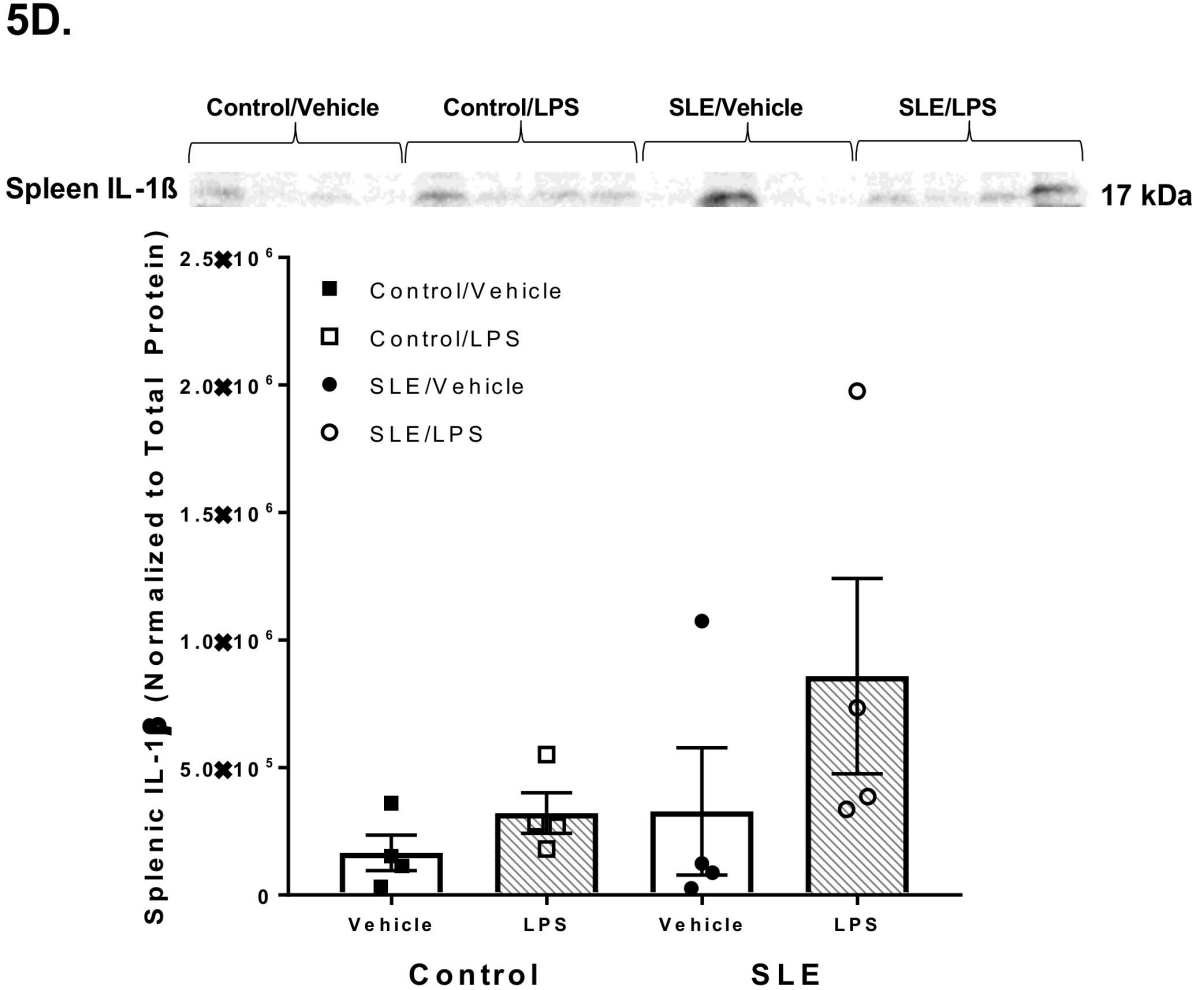

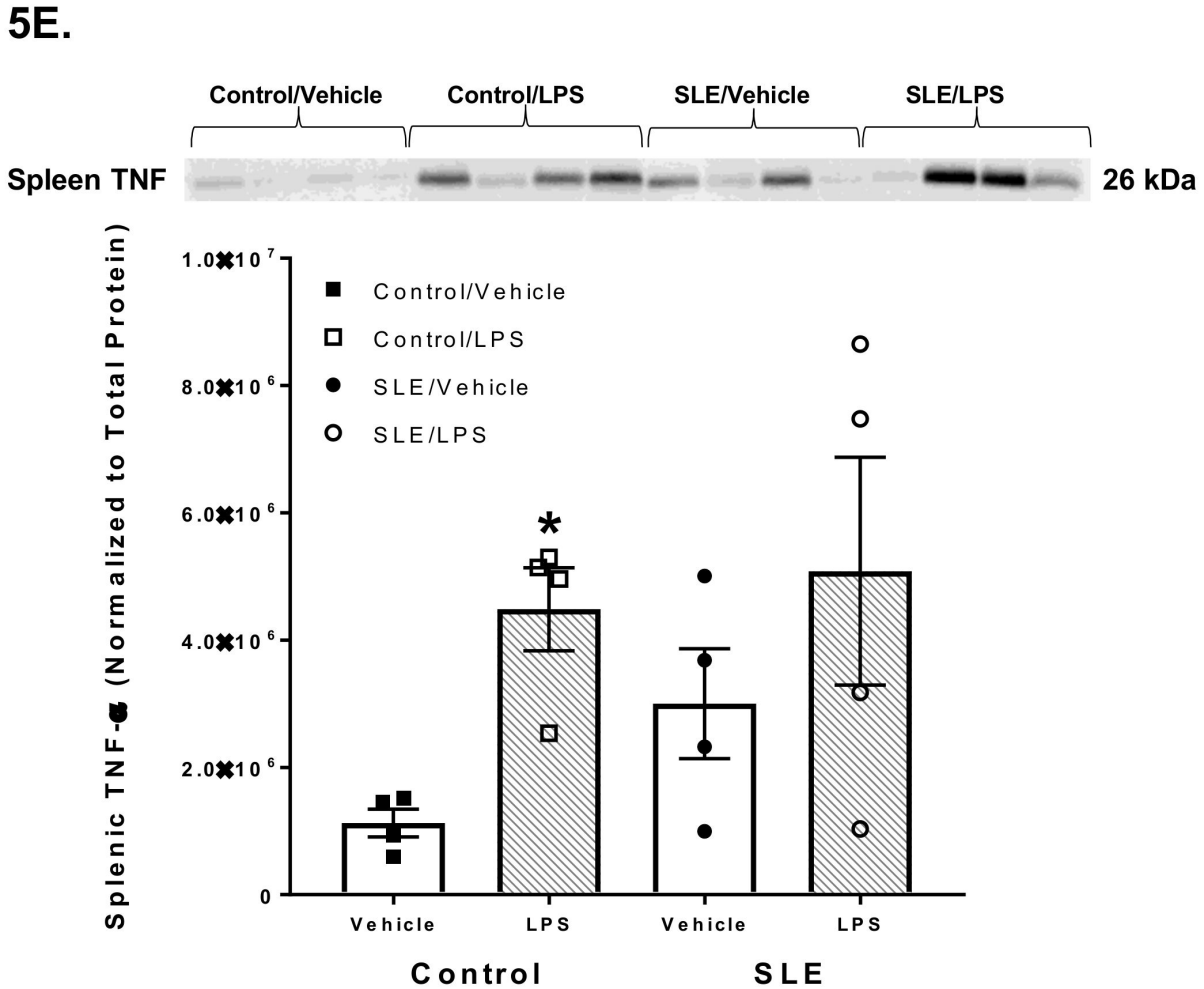

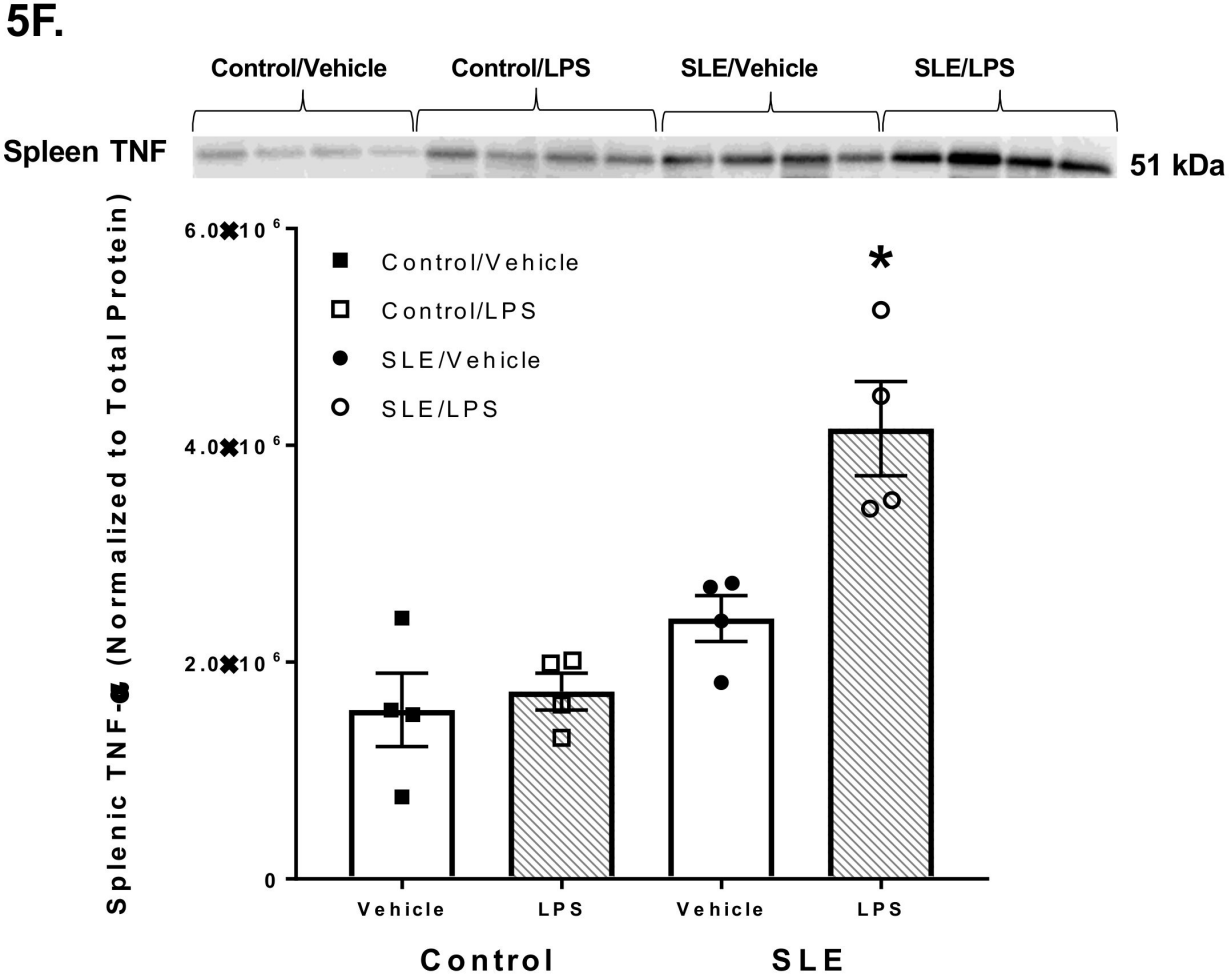
Pro-inflammatory cytokine expression was measured in brains and spleens of lipopolysaccharide (LPS)-challenged and vehicle-treated systemic lupus erythematosus (SLE) and control (*NZW*) mice. (**A**) Brain IL-1β was elevated in SLE mice compared to controls. LPS-challenged controls had elevated brain IL-1β compared to their vehicle-treated counterparts. (**B**, **C**) Brain TNF-α (both 26 and 51 kDa) did not differ between SLE and control mice. LPS-challenged SLE mice had higher TNF-α than vehicle-treated SLE mice. This elevation was not seen in control mice. (**D**) Splenic IL-1β did not significantly differ between any groups of mice. (**E**, **F**) Splenic TNF-α did not differ between SLE and control mice. The 26 kDa isoform of splenic TNF-α was elevated following LPS challenge in control mice when compared their vehicle-treated counterparts, but this was not observed in SLE mice. Contrastingly, the 51 kDa isoform of splenic TNF-α was increased in LPS-challenged SLE mice compared to their vehicle-treated counterparts, and this phenomenon was not seen in control mice. All values are reported as mean ± SEM, and results from a two-way ANOVA indicate significant differences. * *p* SLE/LPS vs. SLE/Vehicle and Control/LPS vs. Control/Vehicle; + *p* SLE/Vehicle vs. Control/Vehicle.

Brain IL-1β was elevated in SLE mice compared to controls (9.2 × 10^5^ ± 1.5 × 10^5^ vs. 3.4 × 10^5^ ± 1.6 × 10^4^, *p* = 0.009) (Figure 5A). LPS-challenged control mice had elevated brain IL-1β compared to vehicle-treated controls (1.0 × 10^6^ ± 1.9 × 10^5^ vs. 3.4 × 10^5^ ± 1.6 × 10^4^; *p* = 0.004). However, LPS challenge did not significantly alter brain IL-1β in SLE mice, although there was a higher trend than vehicle-treated SLE mice. (1.3 × 10^6^ ± 1.1 × 10^5^ vs. 9.2 × 10^5^ ± 1.5 × 10^5^; *p* = 0.062).

Brain TNF-α (both 26 and 51 kDa isoforms) did not differ between SLE and control mice (1.2 × 10^6^ ± 6.1 × 10^5^ vs. 3.4 × 10^5^ ± 7.6 × 10^4^ intensity units, *p* = 0.754 for 26 kDa; 6.4 × 10^5^ ± 2.4 × 10^5^ vs. 3.3 × 10^4^ ± 7.9 × 10^3^ intensity units, *p* = 0.766 for 51 kDa) (Figure 5B,C). LPS-challenged SLE mice had higher 51 kDa brain TNF-α expression (5.1 × 10^6^ ± 2.8 × 10^6^ vs. 6.4 × 10^5^ ± 2.4 × 10^5^ intensity units, *p* = 0.044 for 51 kDa), but not 26 kDa (6.6 × 10^6^ ± 3.9 × 10^6^ vs. 1.2 × 10^6^ ± 6.1 × 10^5^ intensity units, *p* = 0.083 for 26 kDa), compared to their vehicle-treated counterparts. Curiously, LPS challenge did not result in differences in brain TNF-α in control mice (2.6 × 10^5^ ± 1.1 × 10^5^ vs. 3.4 × 10^5^ ± 7.6 × 10^4^ intensity units, *p* = 0.978 for 26 kDa; 1.1 × 10^5^ ± 2.4 × 10^4^ vs. 3.3 × 10^4^ ± 7.9 × 10^3^ intensity units, *p* = 0.968 for 51 kDa). Lastly, LPS-challenged SLE mice had increased brain TNF-α compared to LPS-challenged control mice (6.6 × 10^6^ ± 3.9 × 10^6^ vs. 2.6 × 10^5^ ± 1.1 × 10^5^ intensity units, *p* = 0.045 for 26 kDa; 5.1 × 10^6^ ± 2.8 × 10^6^ vs. 1.1 × 10^5^ ± 2.4 × 10^4^ intensity units, *p* = 0.027 for 51 kDa).

Splenic IL-1β did not differ between SLE and control mice (3.3 × 10^5^ ± 2.5 × 10^5^ vs. 1.7 × 10^5^ ± 7.0 × 10^4^ intensity units, *p* = 0.633) (Figure 5D). LPS challenge did not affect splenic IL-1β in SLE (8.6 × 10^5^ ± 3.8 × 10^5^ vs. 3.3 × 10^5^ ± 2.5 × 10^5^ intensity units, *p* = 0.136) nor control (3.2 × 10^5^ ± 8.0 × 10^4^ vs. 1.7 × 10^5^ ± 7.0 × 10^4^ intensity units, *p* = 0.646) mice. Splenic TNF-α (26 and 51 kDa) did not differ between vehicle-treated SLE and control mice (3.0 × 10^6^ ± 8.6 × 10^5^ vs. 1.1 × 10^6^ ± 2.2 × 10^5^ intensity units, *p* = 0.231 for 26 kDa; 2.4 × 10^6^ ± 2.1 × 10^5^ vs. 1.6 × 10^6^ ± 3.4 × 10^5^ intensity units, *p* = 0.076 for 51 kDa) (Figure 5E,F). The 26 kDa form of splenic TNF-α was not significantly elevated in LPS-challenged SLE mice compared to their counterparts (5.1 × 10^6^ ± 1.8 × 10^5^ vs. 3.0 × 10^6^ ± 8.6 × 10^5^ intensity units, *p* = 0.187), however, was increased in LPS-challenged controls compared to vehicle-treated controls (4.5 × 10^6^ ± 6.5 × 10^5^ vs. 1.1 × 10^6^ ± 2.2 × 10^5^ intensity units, *p* = 0.043). Contrastingly, the 51 kDa form of splenic TNF-α was elevated in SLE mice compared to their vehicle-treated counterparts following LPS challenge (4.2 × 10^6^ ± 4.3 × 10^5^ vs. 2.4 × 10^6^ ± 2.1 × 10^5^ intensity units, *p* = 0.002), while there was no change between LPS-challenged and vehicle-treated controls (1.7 × 10^6^ ± 1.7 × 10^5^ vs. 1.6 × 10^6^ ± 3.4 × 10^5^ intensity units, *p* = 0.705).

## 4. Discussion

The HPA axis is intriguing in conditions of chronic inflammation because, despite its known powerful ability to counteract inflammation, this neuro-endocrine-immune pathway is not always effective. This is the case in the chronic autoimmune inflammatory disorder SLE, and it is unknown whether the dysfunction of the HPA axis occurs at the level of the afferent vagal-hypothalamic PVN interface, the anterior pituitary, or the adrenals. We examined this important question by monitoring innate immune responses in the brain and spleen following an acute inflammatory stimulus (i.e., LPS injection). We found that, following an acute LPS challenge, (1) c-Fos expression in the PVN was similar between SLE and control mice, (2) plasma ACTH was not altered in SLE or control mice, (3) SLE mice had an increased plasma corticosterone whereas controls did not, and (4) brain and spleen TNF-α was increased in SLE mice compared to their vehicle-treated counterparts.

SLE remains a major health disparity and disease burden worldwide. Recent epidemiological studies estimate the prevalence of SLE to be 6 to 178 cases per 100,000 worldwide [33]. While the exact etiology of SLE is unknown, current research supports the interaction between genetic and environmental factors that culminates in a loss of self-tolerance and over-activation of the adaptive immune, specifically T cells and B cells [34]. All organ systems may be damaged in SLE and this is typically the result of inflammatory immune complexes formed from pathogenic autoantibodies (anti-dsDNA being the most diagnostic and prognostic) because of immune cell over-activation [35,36]. As chronic inflammation mediates end-organ damage, treatment is focused upon preventing disease flares and suppressing the immune system, which is mainly achieved through exogenous glucocorticoids and immunomodulatory agents that suppress specific immune cell populations or immune processes [37]. 

A large body of research investigating HPA axis dysfunction in SLE and other autoimmune diseases had taken place during the late 1990s and early 2000s, but inquiry within this niche has since dwindled. It is difficult to elucidate whether HPA axis dysfunction occurs before the onset of rheumatic diseases, but the chronic inflammatory burden in autoimmune diseases does impair the HPA axis [38]. The exact etiology for HPA axis dysfunction in SLE is not known, but may be the result of antibody-mediated neuroinflammation, as others have found that autoantibodies mediate neuropsychiatric disease presentation in the NZM88 murine model of SLE [39]. Contemporary biomedical research in autoimmune diseases is more focused towards immunosuppressive and immunomodulatory agents that target specific immune cell populations or proteins implicated in disease maintenance. There are multiple challenges in elucidating disease mechanisms involving the HPA in autoimmune diseases: (a) both human patients and experimental animal models present with highly variable disease phenotypes, (b) human patients are likely to be on exogenous corticosteroids or immunomodulatory drugs, and (c) the time courses for both HPA axis development and SLE disease progression may vary with other external factors, such as timing of maternal separation [40]. However, as a major endogenous regulator of systemic inflammation, the HPA axis and its characteristic dysfunction in autoimmune diseases are worth thorough comprehension. We utilized LPS as a means of gauging HPA axis dysfunction because we were specifically interested in the role of vagal afferents in transmitting information about peripheral inflammation centrally, and others have established that LPS activates the HPA axis through vagal afferents [41]. Insulin-induced hypoglycemic challenge has also been used to investigate HPA axis dysfunction in murine SLE [16,17]. However, because the *NZBWF1* strain presents with features of metabolic syndrome and we did not want this characteristic to also affect plasma corticosterone and because our interest remains with that of the vagus nerve’s role in HPA axis dysfunction, we used LPS in this study [42].

The finding that c-Fos expression in the hypothalamic parvocellular neurons was comparable between SLE mice and control strains indicates that the afferent vagal nerve-PVN interaction may not be compromised in SLE (Figure 3C). Likewise, no differences in plasma ACTH were observed between SLE and control mice (Figure 4A). Clinical studies have been mixed as to whether basal serum concentrations of cortisol, an anti-inflammatory steroid hormone, are lower in patients with SLE [19]. In our study we did not observe decreased plasma corticosterone in our unchallenged SLE mice compared to control strains (Figure 4B). Other animal studies support these data by demonstrating a diminished ability to raise plasma corticosterone compared to controls following insulin-induced hypoglycemia [18]. Conversely, in the current study, we found there was a greater increase in plasma corticosterone in SLE mice compared to controls following a challenge with a different stressor. The dose chosen for LPS challenge (1 mg/kg body weight [29]) was not sufficient to elevate plasma corticosterone in control mice relative to vehicle-treated controls, nor plasma ACTH in both strains of mice (Figure 4A). However, SLE mice that had received LPS had elevated plasma corticosterone compared to vehicle-treated SLE mice (Figure 4B). This finding was contrary to our anticipated outcome, that the same dosage of LPS would result in a lesser increase of plasma corticosterone in SLE mice compared to controls. This could be due to different processing of inflammatory stimuli in experimental SLE compared to wild-type mice, especially in light of chronically elevated peripheral inflammation in SLE. Additionally, an in vitro study by Klein et al. demonstrated that lymphocytes isolated from SLE patients catabolize cortisol at a decreased rate compared to lymphocytes from healthy subjects [43]. Perhaps this may also be the case concerning lymphocytes and adaptive immune cells in SLE mice, and may help explain the increased plasma corticosterone in SLE mice compared to controls.

Animal studies using wild-type rats have demonstrated that plasma TNF-α decreases significantly following LPS administration and within 2 h of peak plasma corticosterone concentration, so we expected similar responses in our similarly timed study [44]. However, both brain and spleen pro-inflammatory cytokines were elevated in SLE mice that had received LPS (Figure 5A–D) despite increased plasma corticosterone. The heightened peripheral and central inflammation, as well as the increase in plasma corticosterone provoked by LPS, suggest that the systemic inflammatory response, as well as the HPA axis response, may be more sensitive and easily exacerbated in SLE. Perhaps the magnitude of the corticosterone increase elicited by LPS challenge was not sufficient to decrease peripheral inflammation in SLE mice. Alternatively, it may be that glucocorticoid receptor desensitization occurs more frequently in SLE. This latter phenomenon makes sense in light of SLE patients that experience glucocorticoid receptor desensitization as a result of exogenous glucocorticoid therapy. Additionally, glucocorticoid receptor polymorphisms have also been associated with SLE in certain populations, which suggests that glucocorticoid receptor desensitization could be a feature of SLE [45,46]. As in SLE patients, endogenous mechanisms of resistance to glucocorticoids operate in SLE mice and the augmented production of endogenous corticosterone in response to LPS is a compensatory, though ineffective, attempt [47]. Interestingly, resistance to both endogenous and exogenous glucocorticoids is primarily induced by the pro-inflammatory cytokine macrophage migration inhibitory factor (MIF) that plays a role in animal models of autoimmune disease, such as multiple sclerosis, Guillain-Barre syndrome, and SLE [48,49,50]. It is of particular relevance in the context of this paper that *NZBWF1* SLE-prone mice have elevated expression of molecules of the CD74/MIF pathway on B cells and in two target organs, namely, brain hippocampi and kidneys of SLE-afflicted mice, which may also contribute to corticosteroid resistance in SLE [50].

Exogenous glucocorticoids emerged in the 1950s, and have remained a mainstay in SLE treatment [51]. However, synthetic glucocorticoids have multiple unwanted side effects, most commonly, weight gain, but also severe side effects, like compression fractures and psychosis [52]. Prior to the introduction of synthetic glucocorticoids, such as prednisone, ACTH and cortisone were the standard of treatment [53]. Recently, ACTH-containing gels and supplements have re-emerged as an alternative for patients who cannot tolerate glucocorticoids [54]. As an active agent, ACTH exerts direct anti-inflammatory effects directly through melanocortin (M)-1-5 receptors, which leads to nuclear factor kappa B inhibition, as well as indirect effects via stimulation of cortisol release [55]. While contemporary pharmaceuticals have shifted towards B cell depletion and other B cell-directed modalities, the HPA axis continues to be relevant in the treatment of severe SLE and synthetic hormones targeting the HPA axis have greatly decreased morbidity and mortality since their widespread use [56]. For these reasons, studies like ours help to clearly define nuances in HPA axis dysfunction, which could assist with more effective drug regimens.

Vagus nerve stimulation (VNS) has also been indicated for autoimmune diseases, as it activates the cholinergic anti-inflammatory pathway [57,58,59,60,61]. However, others have shown that electrical vagus nerve stimulation can activate afferent vagal fibers and directly prompt HPA axis activity [21]. Since 80% of vagal nerve fibers are afferent, and it is difficult to selectively stimulate afferent or efferent fibers, we posit that electrical vagus nerve stimulators will lead to afferent vagal fiber conduction and increased HPA axis activity, although current studies and clinical trials have not focused on validating the role of the HPA axis in VNS’s therapeutic effects.

In summary, our findings that LPS challenge increases plasma corticosterone in SLE mice, yet results in greater brain and spleen inflammation, appear to confirm our hypothesis that, in SLE, HPA axis activity may not be adequate to combat inflammation. The existing research into HPA axis dysfunction in SLE and other autoimmune diseases is fraught with variation stemming from clinical populations, experimental animal models, medication status, and disease flares. Our current studies have successfully brought attention to the phenomenon of HPA axis dysfunction at the level of effector glands and hormones, and future studies should focus on enhanced sensitivity of corticosterone release and corticosterone receptor sensitization, and the effect this has in chronic inflammatory conditions like SLE.

## Reference
